# Isoprene: An Antioxidant Itself or a Molecule with Multiple Regulatory Functions in Plants?

**DOI:** 10.3390/antiox10050684

**Published:** 2021-04-27

**Authors:** Susanna Pollastri, Ivan Baccelli, Francesco Loreto

**Affiliations:** 1 Institute for Sustainable Plant Protection (IPSP), The National Research Council of Italy (CNR), Via Madonna del Piano 10, 50019 Sesto Fiorentino (FI), Italy; 2 Department of Biology, The University of Naples Federico II, Via Cinthia, 80126 Naples, Italy

**Keywords:** isoprenoids, reactive oxygen species (ROS), defense priming, signaling, hormones, volatile organic compounds (VOCs)

## Abstract

Isoprene (C_5_H_8_) is a small lipophilic, volatile organic compound (VOC), synthesized in chloroplasts of plants through the photosynthesis-dependent 2-C-methyl-D-erythritol 4-phosphate (MEP) pathway. Isoprene-emitting plants are better protected against thermal and oxidative stresses but only about 20% of the terrestrial plants are able to synthesize isoprene. Many studies have been performed to understand the still elusive isoprene protective mechanism. Isoprene reacts with, and quenches, many harmful reactive oxygen species (ROS) like singlet oxygen (^1^O_2_). A role for isoprene as antioxidant, made possible by its reduced state and conjugated double bonds, has been often suggested, and sometimes demonstrated. However, as isoprene is present at very low concentrations compared to other molecules, its antioxidant role is still controversial. Here we review updated evidences on the function(s) of isoprene, and outline contrasting indications on whether isoprene is an antioxidant directly scavenging ROS, or a membrane strengthener, or a modulator of genomic, proteomic and metabolomic profiles (perhaps as a secondary effect of ROS removal) eventually leading to priming of antioxidant plant defenses, or a signal of stress for neighbor plants alike other VOCs, or a hormone-like molecule, controlling the metabolic flux of other hormones made by the MEP pathway, or acting itself as a growth and development hormone.

## 1. Isoprene: Some Generalities

Isoprene (C_5_H_8_) is a small lipophilic volatile molecule synthesized at the chloroplast level by the 2-C-methyl-D-erythritol 4-phosphate (MEP) pathway [1]. Synthesis of isoprene is costly, both in terms of carbon and energy, as it is estimated that 14 NADPH and 21 ATP are requested to make one isoprene molecule [1]. Isoprene synthase (IspS) is the enzyme that catalyzes the last step of isoprene synthesis converting dimethylallyl diphosphate (DMADP) into isoprene [2]. Besides forming the hemiterpene isoprene, DMADP is also the substrate for the synthesis of some other important isoprenoid molecules like monoterepenes [3], pigments (e.g., carotenoids, chlorophyll prenyl chains, [4]) and hormones (e.g., cytokinins, abscisic acid (ABA), [5]).

In higher plants, isoprene synthesis is light-dependent [6,7]. However, the notion that all photosynthetic organisms make isoprene only in the light is challenged by the recent discovery that algae can produce and emit isoprene heterotrophically [8]. Mixotrophic and heterotrophic production of isoprene via the MEP pathway was earlier reported in bacteria [9], and is known to occur in non-photosynthetic organisms such as animals [10]. Isoprene emission is strongly temperature-sensitive. The evaporation temperature of isoprene is very low (34 °C), hence its prevalent volatile nature. This and the high Q_10_ of IspS explains the exponential growth of isoprene synthesis and emission at physiological temperatures [6].

Due to the presence of conjugated double bounds, isoprene may react very rapidly with many reactive chemical species [11] mainly forming methyl vinyl ketone (MVK) and methacrolein (MAC). These compounds are toxic and rapidly scavenged inside plants [12] which suggests their detoxification [13,14].

The concentration of isoprene in the intercellular airspaces of highly emitting leaves has been estimated at 20–60 μL L^−1^ [15,16], whereas isoprene concentration in thylakoid membranes appears to be very small [17,18]. The simulated daily isoprene emission per average leaf during summer is around 1–2 mg C m^−2^ leaf d^−1^ [19] and the amount of isoprene released into the atmosphere is around 500–600 Tg C yr^−1^ at global level [20,21].

**Figure 1:**
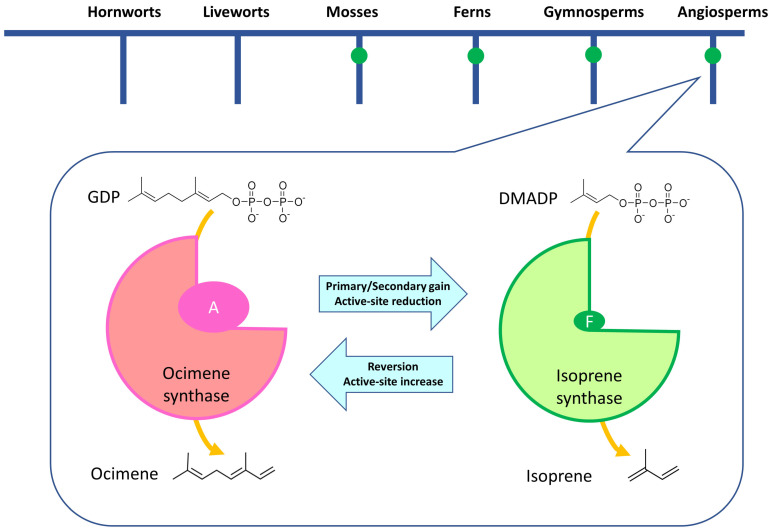
The capacity to emit isoprene (represented by green dots on the different evolutionary lineages) has evolved multiple times in higher plants and in their ancestors. In angiosperms, a model of gain and loss of isoprene synthase function has been proposed [24]. Figure inset readapted from [24] represents the specific step. Ocimene synthase converts geranyl pyrophosphate (GDP) in ocimene whereas isoprene synthase is able to generate isoprene using dimethylallyl pyrophosphate (DMADP) as a precursor. Which enzyme is effectively used depends on the size of the active site, which is increased in the case of ocimene synthase due to the presence of alanine (A) and reduced in the case of isoprene synthase due to the presence of phenylalanine (F). Substitution of A with F has repeatedly occurred over evolution leading to primary gain of isoprene emission capacity, but reversion from F to A has also occurred repeatedly, leading to loss of isoprene emission capacity.

Not all plants emit isoprene. According to Loreto and Fineschi [22] only about 20% of the plant species sampled across biomes worldwide emit considerably amount of isoprene, although other estimates show a larger number of emitters in the tropics. Isoprene-emitting plants are mainly perennial and with no clear phylogenetic thread. The capacity to emit isoprene may have appeared multiple times during evolution of the different lineages of plants [23] (Figure 1). Isoprene synthase seems to be neo-functionalized by monoterpenes, especially ocimene. A secondary gain of IspS from ocimene synthase (OciS) because of a single amino acid mutation has been demonstrated (Figure 1), whereas a primary gain from ocimene is still only hypothesized [24]. While neo-functionalization of isoprene was demonstrated in angiosperms, it might have also occurred in gymnosperms as plants of the two divisions share the same amino acidic sequence where the single mutation leading to isoprene was retrieved [24].

More than half a century after the discovery of isoprene emission by plants [25,26], unclear evolution and uneven pattern of emission have not helped determine a physiological function for isoprene. Among the volatile isoprenoids made by MEP, monoterpenes have clear roles in plant defense and communication with other organisms [27,28]. However, function(s) of isoprene seem to be more elusive [29]. Here we review research about isoprene functions, from earlier studies showing antioxidant properties to more recent results prompting a priming action and even a hormone-like role for isoprene.

## 2. Isoprene Protection from Abiotic Stress

Isoprene has a recognized role in protecting plants against many abiotic stresses. Isoprene-emitting plants are better protected against heat stress [30,31,32,33], drought [11,34], oxidative stress like ozone and other reactive oxygen species (ROS). It has been shown that isoprene is able to quench ozone [35], hydrogen peroxide [36], singlet oxygen [37,38], and nitric oxide [39]. The ability of isoprene to act as a ROS scavenger has led to hypothesize a direct antioxidant role for this molecule, but whether isoprene scavenges ROS inside leaves or in the leaf boundary layer after exiting leaves through stomata remains unclear. Isoprene reaction with ROS inside leaves was indirectly assessed by measuring growing emissions of MVK and MAC from leaves of isoprene-emitting plants exposed to increasingly high temperatures [40]. However, it is now demonstrated that these secondary oxidants may be formed inside leaves from sources other than isoprene [14].

Isoprene-emitting plants are able to maintain high photosynthetic rates for longer periods when exposed to abiotic stresses. This implies that isoprene directly protects the photosynthetic apparatus. Indeed, it has been shown that isoprene improves thylakoid membrane stability [41,42] beside quenching ROS [35,36,43].

Many studies have been performed to find out the specific mechanism by which isoprene exerts its protection on photosynthesis. Chloroplast thylakoid membranes of isoprene-emitting plants are more resistant to denaturation. The main evidence for this is that the non-photochemical quenching of chlorophyll fluorescence (NPQ), which estimates the excess of light dissipated as heat, increases less in isoprene-emitting leaves than in leaves that do not produce isoprene, especially under conditions that normally limit photosynthesis. NPQ may remain stable because thylakoids are less challenged by ROS that are scavenged by isoprene before they reach the membranes under stress conditions [44]. However, NPQ is lower in isoprene-emitting plants than in non-emitting plants, even under conditions that allow photosynthesis to run at its best [45]. Thus, there may be additional explanations for the beneficial effect of isoprene. The lipophilic nature of isoprene may help strengthen the lipidic layer of membranes by intercalating inside them [41,46]. However, it has been pointed out that isoprene concentration in the membranes is theoretically too low to cause any significant direct membrane stabilization [17]. Pollastri et al. [45] surmised that the low evaporation temperature may allow isoprene to remove heat from the membrane, which could help stabilize NPQ, and allow better photochemical efficiency. However, also in this case, theoretical calculations have pointed out that this may reduce temperature by a trivial 0.001 °C even at leaf temperatures >34 °C [45].

**Figure 2:**
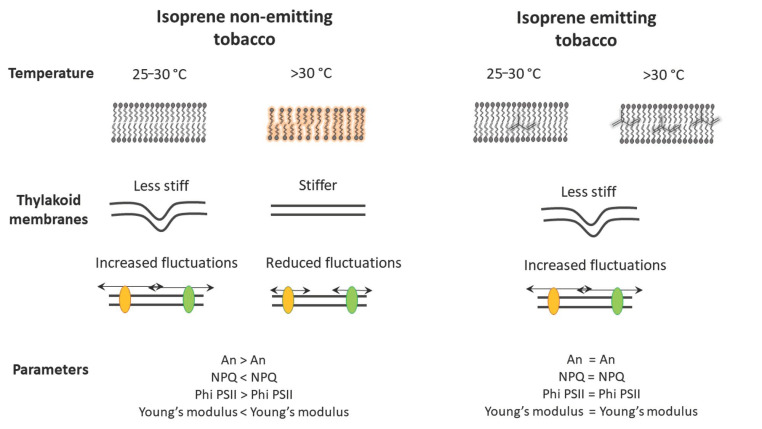
Schematic overview of isoprene mode of action on membrane physiology, according to [47]. At physiological temperatures (25–30 °C), photosynthesis (An), non-photochemical quenching of chlorophyll fluorescence (NPQ), efficiency of Photosystem II (Phi PSII) and thylakoid membrane stiffness (Young’s modulus) are similar in isoprene-emitting and non-emitting leaves. At temperatures higher than 30 °C isoprene-emitters maintain unaltered these parameters, whereas in non-emitters An and PhiPSII were reduced, and NPQ and Young’s modulus increased. These results and further tests of membrane stability are explained by the fact that thylakoidal membranes of isoprene-emitting leaves uphold reduced stiffness even at high temperatures, which maintains the order of membrane lipid chains, and allows increased fluctuations and diffusions between molecules within the membranes, in turn keeping stable photosynthesis.

In recent studies, it has been shown that cells of isoprene-emitting leaves maintain the same elasticity and fluidity at rising temperature at which cells of non-emitting leaves become stiff and loose fluidity [42,47,48]. In particular, Siwko et al. [48] using molecular dynamics simulation techniques, found that isoprene enhances the order of the lipid tails within the membrane mimicking the effect of a reduction of temperature in a dose dependent manner. On the other hand, Velikova et al. [42] indirectly measured the increased thermostability of thylakoid membranes in isoprene-emitting plants with three different biophysical methods, and Pollastri et al. [47] directly measured the stiffness of thylakoid membranes in isoprene-emitting and non-emitting plants at rising temperatures. A low stiffness of the thylakoid membrane, which is inversely correlated with its elasticity, is important for photosynthesis stability and photoprotection [49]. At rising temperatures, the stiffness of isoprene-emitting tobacco thylakoid membranes remains constant, whereas it increases in non-emitting leaves. As a consequence, the thylakoid membranes of isoprene-emitting leaves maintain better conditions for molecular diffusion, electron transport rate, dynamic swelling of the lumen and structural and molecular reorganization under elevated temperatures (Figure 2).

## 3. Isoprene Modulation of Genomic, Proteomic and Metabolomic Profiles

Availability of transgenic plants with induced or repressed isoprene synthesis has allowed to study in depth isoprene-induced changes on the biology of plants from genes to phenotypes. These studies have generally shown that isoprene-emitting plants display different genes expression, and proteomic and metabolomic profiles when compared to non-emitting plants grown under the same conditions. An overview of the results of these studies is reported in Table 1 and briefly reviewed below.

At the gene transcription level, poplars (*Populus* spp.) with repressed levels of the gene encoding for isoprene synthase (*IspS)* showed a reduced expression of genes involved in phenylpropanoids regulatory and biosynthetic pathways, as well as a down regulation of condensed tannins and anthocyanins biosynthetic genes in high light and heat stress conditions [50]. Zuo et al. [51] using *Arabidopsis* and tobacco expressing *IspS* and thus emitting isoprene, as well as non-emitting *Arabidopsis* fumigated with isoprene in unstressed conditions, showed that isoprene alters the expression of genes involved in abiotic and biotic stress defense, growth regulator signaling pathways, photosynthesis, seed germination, and seedling and plant growth. Most of these genes belong to jasmonic acid (JA) mediated defense signaling, phenylpropanoid biosynthesis and regulation, and cytokinin (CK)-mediated wound repair. Similar results were found by Harvey and Sharkey [52] fumigating *Arabidopsis* plants for 24 h with isoprene under non-stressful conditions. This study revealed an induction of chloroplast, phenylpropanoid biosynthetic and translation machinery genes, as well as of transcription factor-enriched gene networks. Another study showed that *Arabidopsis* plants that can emit isoprene because of *IspS* insertion were more sensitive to exogenous ABA supplement, which leads to an upregulation of RD29B, a gene involved in abscisic acid-activated signaling pathway [53]. In contrast, under water-stress, the genes COR15A, whose role is to protect stromal proteins, and P5CS, involved in protection from reactive oxidative species, were both downregulated in isoprene-emitting compared to non-emitting *Arabidopsis* [53]. Isoprene-induced modulations of gene expression and pathway interactions were reviewed in detail by Monson et al. [54], and interpreted by the authors as an indication of isoprene potential role in mediating growth-defense tradeoff (see below).

Whereas many have looked at differences in transcriptomics triggered by isoprene biosynthesis, to our knowledge two studies were performed to look at how the capacity to produce isoprene affects proteomics. The study of Velikova et al. [55] specifically addressed chloroplast proteins revealing that transgenic poplars where isoprene emission was inhibited, showed a down-regulation of chloroplast proteins involved in photosynthesis, light reactions, redox regulation, and oxidative stress defense and general metabolism with respect to naturally isoprene-emitting poplars. A recent proteomic study showed a different protein pattern between isoprene-emitting and non-emitting poplars in the field [56]. In particular, in non-emitting plants proteins involved in phenylpropanoid and flavonoid biosynthesis were less abundant, and proteins involved in biosynthesis of carotenoids and jasmonic acid were more abundant than in isoprene-emitting plants, again indicating a reprogramming of the whole secondary metabolism and a possible trade-off within metabolites synthesized by the MEP pathway (isoprene vs. carotenoids). The latter is controversial, based on experimental results, as further discussed below.

Finally, consequences of isoprene emission on the plant metabolome have been often investigated. [50] showed that down-regulation of secondary metabolism genes (see above) was associated to a reduced concentration of total phenolics and condensed tannins in poplar plants where isoprene synthesis was repressed, especially under high temperatures and high light intensities. Photosynthetic pigments and secondary metabolites also seem to be affected by the presence of isoprene. Zuo et al. [51] reported that isoprene-emitting transgenics of *Arabidopsis* and tobacco (*Nicotiana tabacum* L.) have higher contents of chlorophylls and carotenoids than non-emitting wild types. In poplars that naturally emit isoprene, the contents of carotenes, xanthophylls and chlorophylls are higher than in non-emitting transgenic lines, both in light and dark conditions [57]. Behnke et al. [50,58] showed a higher chlorophyll content in isoprene-emitting than in non-emitting poplars in unstressed conditions, whereas carotenoids were not affected by isoprene presence. This was a surprising result, as isoprene and carotenoids are synthesized by the same MEP pathway. However, in response to heat, non-emitting poplars accumulated more zeaxanthin than isoprene-emitting poplars [59], implying a possible trade-off between isoprene and carotenoids. On the other hand, in drought conditions, tobacco transgenic plants that emit isoprene showed more zeaxanthin and antheraxanthin than non-emitting wild types, as well as a higher amount of phenylpropanoids [60]. The lipidic profile also seems to be affected by the presence of isoprene. A reduced amount of monogalactosyldiacylglycerols, digalactosyldiacylglycerols, phospholipids, and unsaturated fatty acids was observed in non-emitting transgenic poplars compared to isoprene-emitting wild types [61].

In summary, in isoprene-emitting plants the potentially higher antioxidant activity at cellular level may not be only due to the presence of isoprene itself, but also to a concurrently enhanced synthesis of other antioxidants (Table 1).

## 4. Isoprene as Hormone Regulating Plant Antioxidant System and Growth

Several plant signaling molecules and phytohormones have isoprenoid origin [62]. Monoterpenes (C_10_), for instance, have been recently demonstrated to contribute to systemic acquired resistance (SAR) to pathogens and participate to plant-to-plant defense-signal propagation [63,64]. Curiously, a hormonal or signaling role for isoprene (C_5_), the building block of isoprenoids, has been overlooked for many years, whereas recently it has become a realistic mode of action of its effects on plants. This line of research gained credit following the observation that *Arabidopsis* plants, which naturally do not emit isoprene, reprogram their transcriptome when exposed to isoprene fumigation [52] (see above).

With the current knowledge, it seems difficult to exclusively consider isoprene as a stress signaling molecule, since various reports relate isoprene to plant growth and development. Recent results obtained in both poplar and *Arabidopsis* plants have shown that *IspS* gene promoter activity takes place in many plant parts, including different root regions such as vascular tissue, root hairs, root cap and developing lateral roots [65]. Promoter activity was stimulated by the treatment with the growth hormone indole-3-acetic acid, and transgenic poplar plants unable to emit isoprene showed increased formation of lateral roots [65]. Isoprene-emitting *Arabidopsis* plants transformed with the *IspS* gene from *Eucalyptus globulus* showed increased hypocotyl, cotyledon, leaf and inflorescence growth, suggesting that isoprene may exert its effects on growth through direct regulation of gene expression [51]. In a different study, isoprene-emitting *Arabidopsis* plants transformed with the *IspS* gene from *Arundo donax* showed reduced growth inhibition after exogenous ABA treatment [53]. On the other hand, negative effects of isoprene on leaf and stem growth were observed in isoprene-emitting tobacco lines. However, an increased hypocotyl growth was observed in these plants, similarly to what occurred in *Arabidopsis* [51]. Therefore, isoprene seems to act as either positive or negative regulator of plant development. Dani et al. [5] proposed a true hormonal effect for isoprene, based on structural identity with chloroplast cytokinins (e.g., zeatin), which are also formed by the MEP pathway. While isoprene is emitted at concentrations far too high to postulate for it a true hormonal action, cooperation between isoprene and cytokinins could be important in controlling leaf development and lifespan ([5], and unpublished results).

It seems reasonable to assume that isoprene can control the antioxidant system and whole plant growth in a hormone-like manner. Notably, the two effects could be related, i.e., isoprene could influence plant development and growth by modulating ROS levels [65]. Interestingly, *IspS* promoter activity was identified in the same root regions where transgenic poplars that do not emit isoprene showed accumulation of ROS, leading the authors to conclude that isoprene can impair both ROS and lateral root growth in poplar roots [65]. This conclusion was corroborated by previous studies showing higher nitric oxide (NO) bursts after ozone stress in leaves in the absence of isoprene emission [39,66], and hydrogen peroxide accumulation under heat and light stress [50,67]. These observations suggest modulation by isoprene of extent and speed at which ROS and NO signaling molecules are generated within plant cells, and an overall role for isoprene as a negative regulator of ROS production. Recent data have suggested how isoprene could exert this effect. RNA-seq data have highlighted isoprene up-regulation of genes encoding molecules with antioxidant activity, genes involved in ROS homeostasis and protection against oxidative stress, and down-regulation of genes involved in ROS accumulation [51]. Similar evidences emerged with proteomic analyses performed in poplar roots [65].

## 5. Isoprene as Defense Priming Molecule: Emerging Clues

As reported above, some of the protective effects of isoprene against abiotic stresses can be explained by considering isoprene as a hormone-like molecule, which can act at the gene level and regulate transcripts and proteins of the antioxidant system of the plant, thereby controlling ROS production and ROS-related biological processes, such as development. However, it has also been hypothesized that isoprene may act as a defense priming signal [51]. In accordance with a priming role for isoprene, Monson et al. [54] have recently outlined an hypothesis according to which isoprene would provide an advantage of better tolerating the stresses by allowing plants to accommodate allocations to defense with less costs to growth. Defense priming is a biological phenomenon that allows plants to increase their resistance/tolerance capacity by enhancing sensitivity and responsiveness to stress [68]. Defense priming is generally accompanied by some key features that can be detected after appropriate stimulation, such as a memory effect (i.e., maintenance of the primed state for a certain period of time after priming stimulation), low fitness costs caused by the priming stimulus compared with the direct activation of defense, broad spectrum protection (protection against different stressors), and especially more robust defensive behavior upon stress occurrence (i.e., stronger/quicker activation of defense responses) with concomitant gain of fitness for the plant when grown in hostile environments [69]. Importantly, some phytohormones have actually been shown to possess priming ability [68,70].

Concerning isoprene, most of the above-mentioned features typical of priming molecules have not been experimentally demonstrated so far, and the capacity to act on the transcriptome (and more recently on proteomes) is not sufficient by itself to prove the establishment of a primed state [71]. Early unpublished experiments suggested a priming effect of isoprene, as isoprene-emitting plants showed higher activities of enzymatic antioxidants and higher levels of heat shock factors and proteins ahead of a heat stress from which they were then more protected than non-emitters (Loreto et al. personal communication). A very recent report seems to confirm a sort of priming effect for isoprene in transgenic *Arabidopsis* lines overexpressing an isoprene synthase gene [53]. In particular, these transgenic, isoprene-emitting lines were found to reduce water loss, enhance ABA-induced stomatal closure, and enhance the expression of the ABA-responsive gene *RD29B* after ABA treatment [53]. In summary, isoprene-emitting lines were more tolerant to dehydration and heat stress by acquiring an enhanced responsiveness to ABA [53]. This pattern is actually reminiscent of priming. For example, the well-known priming molecule β-aminobutyric acid (BABA) has been reported to exert its priming effect in *Arabidopsis* and wheat plants against water stress by potentiating ABA signaling thereby allowing faster decrease of stomatal conductance [72]. ABA is an end product of the MEP pathway, and an enhanced isoprene emission might mark enhanced flux of carbon throughout the overall MEP pathway and be correlated to a higher synthesis of ABA [73].

Another very recent study has also highlighted the ability of isoprene to induce resistance against microbial pathogens [74]. *Arabidopsis* plants exposed for 3 days to the isoprene presence in the surrounding gas phase became more resistant to the infection with the bacterium *Pseudomonas syringae* pv. *tomato* (*Pst*) when subsequently infected [74]. Resistance was successfully induced only in plants possessing functional salicylic acid (SA)-/SAR-signaling genes, such as *NPR1* and *ALD1* genes, demonstrating that isoprene can induce resistance by activating or by priming SA-dependent defenses [74]. Interestingly, induced resistance was still visible 4 days after the end of the exposure, reinforcing the idea of a memory effect caused by priming [74]. In addition, the proximity of isoprene-emitting poplar trees in open air (up to 80 cm far away in greenhouse conditions) was still able to induce resistance to *Pst* infection in *Arabidopsis*, highlighting the potential biological relevance of these new findings and countering the idea that isoprene is not active in plant-plant communication because of its high dilution in the air [18].

In the light of these recent reports it appears now crucial to specifically address the capacity of isoprene to prime tolerance or resistance mechanisms against abiotic or biotic stressors, both in the producing plant as suggested by transgenic *Arabidopsis* lines overexpressing an isoprene synthase gene [65] and in plant-to-plant communication [63].

## 6. Conclusions

We have reviewed literature that attributed to isoprene emitted by plants different functions. Isoprene emission seems to be beneficial for plants, but studies originally indicating a mere antioxidant action, are now revealing an increasingly complex network of processes and mechanisms that are influenced by isoprene. This simple volatile compound which can easily diffuse in plants and out of them, in the surrounding atmosphere, is emerging as a multitasking and helpful molecule that, besides acting as an antioxidant, also primes defensive pathways, acts as an infochemical and cooperates with hormones (Table 2). The abundant emission of isoprene by plants worldwide is perhaps better explained by such a variety of actions assisting plants throughout their lifespan and when challenged by stresses.

## Figures and Tables

**Table 1:** Summary of studies highlighting differences in gene expression, proteins and metabolites in isoprene-emitting and non-emitting plants under different experimental conditions. Up-regulation or down-regulation of gene transcripts, or higher or lower concentrations of proteins and metabolites in isoprene-emitting plants with respect to the non-emitting ones are shown.

Isoprene Effect	Plant	Genes, Proteins, Metabolites Influenced by Isoprene	Condition	Reference
**Genes**				
Up-regulated	Poplar (WT and Knock-out)	Biosynthetic pathways of condensed tannins, anthocyanins and phenylpropanoids, and regulatory pathways of phenylpropanoids genes.	high light and heat stress	[50]
Up- and down-regulated	*Arabidopsis* and tobacco (WT and *IspS*). *Arabidopsis* (WT) fumigated with isoprene	Abiotic and biotic stress defence, growth regulator signaling pathways, photosynthesis, seed germination, and seedling and plant growth, JA mediated defense signaling, phenylpropanoid biosynthesis and regulation, and cytokinins (CK)-mediated wound repair genes.	non-stressful conditions	[51]
Up-regulated	*Arabidopsis* (WT) fumigated with isoprene	Chloroplast, phenylpropanoid biosynthetic and translation machinery genes, transcription factor-enriched gene network	non-stressful conditions	[52]
Up-regulated	*Arabidopsis* (WT and *IspS*)	*RD29B* gene (abscisic acid-activated signaling pathway)	exogenous ABA supplement	[53]
Down-regulated	*Arabidopsis* (WT and IspS)	*COR15A* gene (stromal proteins protection), and *P5CS* (reactive oxidative species protection)	water-stress	[53]
**Proteins**				
Higher concentrations	Poplar (WT and Knockout)	Chloroplast proteins involved in photosynthesis, light reactions, redox regulation, and oxidative stress defense and general metabolism	Control	[55]
Higher concentrations	Poplar (WT and Knockout)	Proteins involved in phenylpropanoid pathway and flavonoid biosynthesis	Control	[56]
Lower concentrations	Poplar (WT and Knockout)	Proteins involved in the biosynthesis of terpenoids, carotenoids and in the methylerythritol phosphate (MEP) and jasmonic acid pathways	Control	[56]
**Metabolites**				
Higher concentrations	Poplar (WT and Knockout)	Total phenolics and condensed tannins	high light and heat stress	[50]
Similar concentrations	Poplar (WT and Knockout)	Photosynthetic pigments and secondary metabolites	high light and heat stress	[50]
Higher concentrations	*Arabidopsis* and tobacco (WT and *IspS*)	Chlorophylls and carotenoids	non-stressful conditions	[51]
Higher concentrations	Poplar (WT and Knockout)	Chlorophylls, carotenes and xanthophylls	light and dark conditions	[57]
Higher concentrations	Poplar (WT and Knockout)	Chlorophyll content	Control	[50,58]
Similar concentrations	Poplar (WT and Knockout)	Carotenoids	Control	[50,58]
Lower concentrations	Poplar (WT and Knockout)	Zeaxanthin	Heat	[59]
Higher concentrations	Tobacco (WT and *IspS*)	Zeaxanthin, anteraxanthin and phenylpropanoids	drought conditions	[60]
Higher concentrations	Poplar (WT and Knockout)	Monogalactosyldiacylglycerols, digalactosyldiacylglycerols, phospholipids and unsaturated fatty acids	Control	[61]

WT, wild type; IspS, Isoprene synthase; RD29B, Responsive to dessication 29B; COR15A, Cold-regulated 15A; P5CS, Pyr-roline-5-carboxylate synthase.

**Table 2:** Summary of all the biological roles that have been proposed for isoprene, with associated functions that are expected to be implemented, and experimental evidence in favor (PRO) or against (CON) the alleged role.

Isoprene Role	Functional Expectations	Experimental Evidence
Antioxidant	Protection against oxidative damage by direct reaction with oxidizing molecules (ROS or NO)	PRO: Isoprene contributes to thermal and oxidative stress tolerance by quenching ROS and reactive nitrogen species [36,39]. CON: Secondary antioxidants generated by reaction with ROS (e.g., methyl vinyl ketone) being per se cytotoxic may be removed without isoprene quenching [14]. Insufficient isopreneconcentration inside membranes to carry out effective antioxidant action [17].
Membrane protection	Stabilization of membrane properties when challenged by stresses by intercalating membrane lipids	PRO: Isoprene lipophylic nature favors isoprene presence in thylakoidal membranes which are kept elastic and fluid at rising temperature [29]. CON: Isoprene concentration in the membranes theoretically very low [17].
Defense priming stimulus	Improving stress resistance/tolerance by boosting stress signal perception, propagation, and/or activation of defense mechanisms. A quicker/stronger defense gene expression during and not before stress conditions is the hallmark of priming.	PRO: Isoprene-emitting transgenic lines show enhanced tolerance to dehydration and heat stress by displaying enhanced ABA-induced stomatal closure, reduced water loss, and higher ABA-induced expression of the ABA-responsive marker gene *RD29B* [53]. Isoprene exposure induces resistance to bacterial infection through SA-dependent defense mechanisms in *Arabidopsis* [74]. Isoprene fumigation induces a small reprogramming of the transcriptome (167 DEGs, fold change >2) in *Arabidopsis*, including up-regulation of some of the most important defensive pathway genes [52]. CON: ROS levels are reduced during thermal and oxidative stress because of direct and rapid antioxidant action of isoprene [36], without any need to invoke priming. Unclear whether isoprene-exposed plants (e.g., *Arabidopsis* in [74]) are more resistant to bacterial infection because of isoprene induction of SA synthesis and activation of SA-dependent defenses ahead of infection (no priming) or because isoprene also primes SA-dependent defenses for a more robust defensive behavior upon infection.
Stress signal	Increased production after stress and role as transmission signal of stresses to other parts of the plant and/or to neighboring plants	PRO: Isoprene emission changes rapidly in response to wounding and temperature [75]. Knocking-down (by RNAi) or inhibiting (by fosmidomycin) isoprene emission negatively impacts thermotolerance [30,33,59]. CON: Isoprene production occurs constitutively in several plants without apparent association to environmental stress [22]. *Isps* gene promoter activity takes place in roots and especially in developing lateral roots [65] during normal growth. Isoprene-emitting transgenic lines show positive effects on growth and development in unstressed plants [51].
Hormone	Regulation, at very low concentration, of plant processes at the gene level	PRO: Isoprene acts as a negative regulator of ROS production by modulating gene expression [51,58,65,66]. Isoprene can both positively and negatively regulate plant growth and development [51,65]. CON: Isoprene is produced and emitted in very large amount by plants [22] which does not make it a hormone by definition.
Infochemical	Attraction of pollinators and insect predators or repulsion of phytophagous insects	PRO: Isoprene is a highly volatile molecule as are many infochemicals [76]. Tobacco plants emitting isoprene repel *Manduca sexta* larvae [77]. The parasitic wasp *Diadegma semiclausum* perceives isoprene by its chemoreceptors and is being repelled [31]. CON: *Chrysomela populi* (poplar leaf beetle) is unable to detect isoprene and show no preference for isoprene-emitting or non-emitting poplar plants [78,79]. Isoprene production occurs constitutively in several plants without apparent association to delivery of infochemical messages [22].

ROS, Reactive oxygen species; NO, Nitric oxide; ABA, Abscisic acid; SA, Salicylic acid; DEGs, Differentially expressed genes; RD29B, Responsive to desiccation 29 B gene; RNAi, RNA interference.
